# Lists of potential diagnoses that final-year medical students need to consider: a modified Delphi study

**DOI:** 10.1186/s12909-021-02652-5

**Published:** 2021-04-23

**Authors:** Yuka Urushibara-Miyachi, Makoto Kikukawa, Masatomi Ikusaka, Junji Otaki, Hiroshi Nishigori

**Affiliations:** 1 Faculty of Medicine, Kyoto University, Yoshida konoe-cho, Sakyo-ku, Kyoto, 606-8501 Japan; 2 Kyushu University, Fukuoka, Japan; 3 Chiba University Hospital, Chiba, Japan; 4 Tokyo Medical University, Tokyo, Japan; 5 Nagoya University, Nagoya, Japan

**Keywords:** Clinical reasoning, Contrastive learning, Undergraduate medical education, Core curriculum, Expert consensus, Modified Delphi approach

## Abstract

**Background:**

Contrastive learning is known to be effective in teaching medical students how to generate diagnostic hypotheses in clinical reasoning. However, there is no international consensus on lists of diagnostic considerations across different medical disciplines regarding the common signs and symptoms that should be learned as part of the undergraduate medical curriculum. In Japan, the national model core curriculum for undergraduate medical education was revised in 2016, and lists of potential diagnoses for 37 common signs, symptoms, and pathophysiology were introduced into the curriculum. This study aimed to validate the list of items based on expert consensus.

**Methods:**

The authors used a modified Delphi method to develop consensus among a panel of 23 expert physician-teachers in clinical reasoning from across Japan. The panel evaluated the items on a 5-point Likert scale, based on whether a disease should be hypothesized by final-year medical students considering given signs, symptoms, or pathophysiology. They also added other diseases that should be hypothesized. A positive consensus was defined as both a 75% rate of panel agreement and a mean of 4 or higher with a standard deviation of less than 1 on the 5-point scale. The study was conducted between September 2017 and March 2018.

**Results:**

This modified Delphi study identified 275 basic and 67 essential other than basic items corresponding to the potential diagnoses for 37 common signs, symptoms, and pathophysiology that Japanese medical students should master before graduation.

**Conclusions:**

The lists developed in the study can be useful for teaching and learning how to generate initial hypotheses by encouraging students’ contrastive learning. Although they were focused on the Japanese educational context, the lists and process of validation are generalizable to other countries for building national consensus on the content of medical education curricula.

**Supplementary Information:**

The online version contains supplementary material available at 10.1186/s12909-021-02652-5.

## Background

Healthcare settings are becoming increasingly complex and the social accountability of physicians in ensuring patient safety is gaining prominence. In this light, medical schools are required to educate and evaluate medical students with sufficiently high standards, so that they become physicians who make the fewest possible diagnostic errors. This is particularly evident in the worldwide trend of incorporating the assessment of clinical reasoning skills in high-stakes examinations such as national medical licensing exams [1]. Thus, greater educational support is required for students to acquire the competence to anticipate a set of differential diagnoses from the earliest phase of the diagnostic process, gather confirming and refuting information according to an initial hypothesis, select and perform the relevant history taking and physical examination, and interpret the findings to confirm or deny the initial hypothesis.

In this context, the lack of development of diagnostic hypotheses remains an issue in the teaching of clinical reasoning to medical students. For example, medical students learn how to take a patient’s history without anticipating differential diagnoses, even though the literature suggests that diagnostic errors can be reduced by querying an initial hypothesis [2]. Furthermore, attempting to diagnose without generating a hypothesis may reduce students’ reasoning performance [3]. Nevertheless, many medical schools have taught physical examination maneuvers in isolation, usually following a systematic “head-to-toe” approach [4]. Recent studies in cognitive load theory have described that such fragmented reasoning may lead to diagnostic errors [5].

How can teachers effectively instruct medical students in hypothesis generation? Schmidt and Mamede [6] found that there is no evidence elucidating the most effective method for teaching clinical reasoning to medical students. In the highly specialized wards of teaching hospitals, which feature increasingly shorter patient stays, patient interaction with and exposure to role model doctors in clinical clerkships may be insufficient for students to gain the competence required in clinical reasoning [7]. This is particularly the case regarding the need to consider the full range of differential diagnoses across all medical disciplines and specialties. Students usually learn specialty-specific or disease-oriented reasoning skills from specialists, who tend to generate diagnostic hypotheses focused on a particular organ system [8]. Variation among the patient cases that students experience tends to be limited, and feedback from attending doctors is opportunistic [6]. Instead, various pre-clinical curricula have been introduced to teach students clinical reasoning, in which cases are usually also not sufficient [6]. In this context, previous research has suggested that a “comparing and contrasting” approach is effective for medical students to foster illness scripts in their minds [6, 9]. Medical students without sufficient clinical experience can effectively formulate an illness script of disease by comparing and contrasting the discriminating clinical features of other competing diagnoses in terms of a particular symptom.

Nevertheless, in the context of undergraduate medical education, there was no learning resource supporting medical students’ contrastive learning by covering a wider variety of signs and symptoms. As of 2017, when this study was conducted, no previous study had developed a consensus on comprehensive lists of differential considerations across different medical disciplines regarding the common signs and symptoms to be learned during the undergraduate medical curriculum. Therefore, this research aimed to develop lists of a limited number of diagnostic considerations regarding all the signs, symptoms, and pathophysiology that medical students should learn before graduation.

These lists, which should consist of diseases of higher priority in terms of clinical importance and urgency, can be universally applicable in the context of undergraduate medical education. However, they may also be specific to the local social context in which they are developed and introduced. The reason for this specificity is that various epidemiological factors and societal needs, which may be variable over time, can influence what diseases medical students learn to diagnose within their countries. Therefore, we decided to focus on the Japanese setting for this study.

In Japan, the fourth version of the national core curriculum for undergraduate medical education in 2016 newly introduced lists of possible diagnoses regarding 37 common signs, symptoms, and pathophysiology (Table 1) that ought to be learned as part of the six-year undergraduate curriculum [10]. An original set of lists was developed through a review of the previous literature on clinical reasoning [11–16] by committee members consisting of general internists and general practitioners specializing in teaching clinical reasoning, which included the authors of this study. It was then followed by a revision based on public feedback. As this process possibly reflected the personal perspectives of a limited number and range of specialists with authority, we attempted to validate the lists by building a consensus among experts in clinical reasoning education through a systematic, iterative process. Thus, the research question for this study asked: what are the potential diagnoses that final-year medical students need to consider for the signs, symptoms, and pathophysiology listed in the national model core curriculum for undergraduate medical education in Japan?

**Table 1:** The 37 common signs, symptoms, and pathophysiology in the national model core curriculum for undergraduate medical education (revised in 2016)

1. Fever	14. Hemosputum/hemoptysis	27. Lymphadenopathy
2. General fatigue	15. Dyspnea	28. Abnormality of urine and urination
3. Appetite loss	16. Chest pain	29. Hematuria/proteinuria
4. Weight gain/loss	17. Palpitation	30. Menstrual disorders
5. Shock	18. Pleural effusion	31. Anxiety/depression
6. Heart arrest	19. Dysphagia	32. Memory loss
7. Disturbance of consciousness/syncope	20. Abdominal pain	33. Headache
8. Seizure	21. Nausea/vomit	34. Motor paralysis
9. Dizziness	22. Hematemesis/melena	35. Back pain
10. Dehydration	23. Constipation/diarrhea	36. Arthralgia/swollen joint
11. Edema	24. Jaundice	37. Trauma/burn
12. Rash	25. Abdominal distension/mass	
13. Cough/sputum	26. Anemia	

## Methods

In this study, we utilized a modified Delphi method [17, 18]. In the original Delphi method [19], an initial list to be examined is established based on feedback from experts in the first round. However, in the modified Delphi method, the initial list is mostly produced by the researchers based on a literature review, interviews with relevant professionals, or other academic methods [20]. The Delphi and modified Delphi methods allow researchers to gather and achieve consensus on the opinions of experts through a series of structured questionnaires, which are conducted anonymously to avoid the influence of authority among the experts [19]. This is considered one of the strengths of these methods, particularly in East-Asian countries such as Japan, where hierarchical social relationships tend to have a strong influence on the stakeholders of decision-making processes [21].

Both methods have been used in a variety of studies to establish a consensus on core competencies or curricula regarding a specific topic or domain among medical specialties. For instance, Alahlafi and Burge [22] conducted a Delphi study to build a consensus on what medical students need to learn about psoriasis. Battistone, Barker, Beck, Tashjian, and Cannon [23] defined the core skills of shoulder and knee assessment using the Delphi method. Moore and Chalk [24] used the Delphi method to produce a consensus on neurological physical examination skills that should be learned by medical students. Finally, Moercke and Eika [25] used the Delphi method to identify the required clinical skills and minimum competency levels therein during undergraduate training.

### The expert panel

Although there is no consensus on the most appropriate number of experts to include in a panel for Delphi studies, previous studies have generally required at least 20 expert participants for sufficient reliability [26]. Considering the average response rate in past Delphi studies of approximately 80% [27], we aimed to recruit 25 participants. We decided to exclusively recruit generalists for this study because it required all-round clinical reasoning competence for a wide range of signs, symptoms, and pathophysiology without being limited or biased to a particular organ or discipline-specific condition or disease.

Our inclusion criteria were: 1) clinical faculty members at Japanese medical schools, who were involved in teaching clinical reasoning to medical students, or 2) clinical teachers at training hospitals in Japan, who had experience in teaching clinical reasoning to medical students at universities.

We have excluded those who: 1) had less than 10 years of experience in the practice of clinical reasoning, 2) were non-physician healthcare professionals, 3) were residents and medical students, or 4) were clinical teachers in training hospitals who did not have teaching experience as university faculty.

Accordingly, we targeted physicians who were recognized as authorities in Japan, such as those who had published books on clinical reasoning education or organized educational opportunities, physicians who were representatives of departments of general practice at universities or training hospitals, and young physicians who were representatives of groups that were providing educational opportunities on clinical reasoning education.

The previous literature also suggested that recruiting a variety of participants may produce higher quality results that are more generally acceptable [21]. Thus, we used purposeful sampling to recruit participants to ensure diversity in terms of gender, age, and geography, as well as affiliations. The four authors (MI, JO, MK, HN), who were general internists specializing in teaching clinical reasoning, produced a list of candidates for discussion, which included two female candidates. YM, who was a physician-researcher on clinical reasoning, contacted all the potential participants via email to ensure their interest and obtain their agreement to participate. Of the potential pool, 23 candidates agreed to join the study, but the two female candidates declined. Thus, we assembled a panel of 23 physicians, and informed consent was obtained from all participants.

### The initial lists

The initial lists regarding the 37 common signs, symptoms, and pathophysiology consisted of 277 items. The model core curriculum ensures core competencies for medical students that contribute to healthcare for the general population. Thus, for this study, which aimed to revise parts of it, the target population was the general population. The sources reviewed to produce the initial lists included both domestic [11, 12] and international [13–16] literature regarding diagnostic considerations related to the 37 signs, symptoms, and pathophysiology.

The original 37 lists of diagnostic considerations in the model core curriculum consisted of the 170 diseases designated as the minimum requirements, named “required basic facts,” in the 2018 version of the guideline of national license examination for physicians [28]. This was because the lists were intended to be only a baseline resource for medical students, which they could use to accumulate new knowledge for making diagnoses. Thus, “required basic facts” in the guideline were also adopted as the rationale for selection of diagnostic considerations for this study to develop the lists consisting of the minimum essential diseases.

The questionnaire, edited by YM, was piloted by two members of the research team (HN and MK) who confirmed that it could be answered in approximately 30 min. We also revised the layout of the questionnaire to make it more readable and to reduce the possible cognitive load on respondents. As previous studies, including Hasson et al. [19], suggested two or three rounds for Delphi studies, we designed our study using a two-round modified Delphi method, with an optional additional third round for re-evaluating certain items, an option that we ended up using. In all rounds, questionnaires with the lists to be evaluated were provided to the participants via a web-based survey system (Google Forms: available at https://www.google.com/intl/ja/forms/about/).

In Round 1, for each list of potential diagnoses for the 37 signs, symptoms, and pathophysiology, the participants were asked to evaluate on a 5-point Likert scale (1 = absolutely needs to be excluded, 5 = absolutely needs to be included) whether each item (i.e., disease) should be hypothesized by final-year medical students when diagnosing a patient with the given signs, symptoms, or pathophysiology. The participants were also asked to add any other diseases they considered relevant to include in the list. Their responses were anonymously collected and analyzed with a predefined standard for positive consensus as follows. Based on our literature review, which included research from Dielissen et al. [27] and Heiko [29], our standard was 1) a mean score of 4 and higher with 2) a standard deviation of less than 1 and 3) 75% or more of the experts scoring 4 or 5 (i.e., 75% agreement). The participants were given two weeks to evaluate the lists in each round. To keep the response rates sufficiently high, the participants received reminder e-mails a few days before each deadline, and incentives were provided for each round. Two researchers (YM and HN) analyzed the primary data and discussed the results with the other researchers. Among all the additional items suggested by the panel, only the diseases listed as “required basic facts” for the national licensing exam were incorporated into the revised list for Round 2.

The second version of the list, to be evaluated in Round 2, consisted of 1) diseases added by the panelists that were part of the “required basic facts” for the national licensing exam, 2) diseases from the initial lists that did not meet our positive consensus standard, as well as 3) diseases from the initial lists that met the standard. Different strategies can be taken after the second round in accordance with the purpose of the study [30]. The authors discussed and agreed that it was necessary for the panelists to review the overall picture of which diseases should be included in the list of possible diagnoses related to signs, symptoms, or pathophysiology, to evaluate the priority of the newly added diseases by the panelists. Thus, we requested the panelists to reevaluate the items that met or did not meet the consensus criteria in the first round, for the purpose of approval for inclusion or exclusion from the list, respectively. We made sure to communicate fully the need for this method when conducting the second round.

The panel provided free-form comments on the questionnaire in every round. The study took place between September 2017 and March 2018 and was approved by the institutional research board of the Kyoto University Graduate School of Medicine (R0481). This study complied with the Strengthening the Reporting of Observational Studies in Epidemiology (STROBE) guidelines [31] and a guide for the conducting and reporting of Delphi studies (CREDES) [30].

## Results

The participating experts, who had 11 years or more (average 21 years) of clinical expertise and more than 5 years (average 13 years) of teaching expertise, were all male from all across Japan. Eight of them had more than two specialties (Table 2).

**Table 2:** Demographics of the expert panel (*n* = 23)

Characteristic	No. (%)
Years in practice	
10–19 years	14 (61)
20–29 years	4 (17)
More than 30 years	5 (22)
Years of involvement in clinical teaching	
Less than 10 years	6 (26)
10–19 years	13 (57)
More than 20 years	4 (17)
Certifications/specialties (Multiple answers allowed)	
General Medicine/General Internal Medicine	20 (88)
Family Medicine	3 (13)
Internal Medicine	2 (9)
Geriatrics	1 (4)
Emergency Medicine	1 (4)
Gastroenterology	1 (4)
Neurology	1 (4)
Cardiology	1 (4)
Medical Education	2 (9)
More than two specialties	8 (35)
Practice/educational settings	
University/University hospital	19 (83)
Community hospital	4 (17)
Regions	
Kanto	9 (39)
Chubu	5 (22)
Kansai	2 (9)
Chugoku	1 (4)
Kyushu	6 (26)

**Fig. 1:**
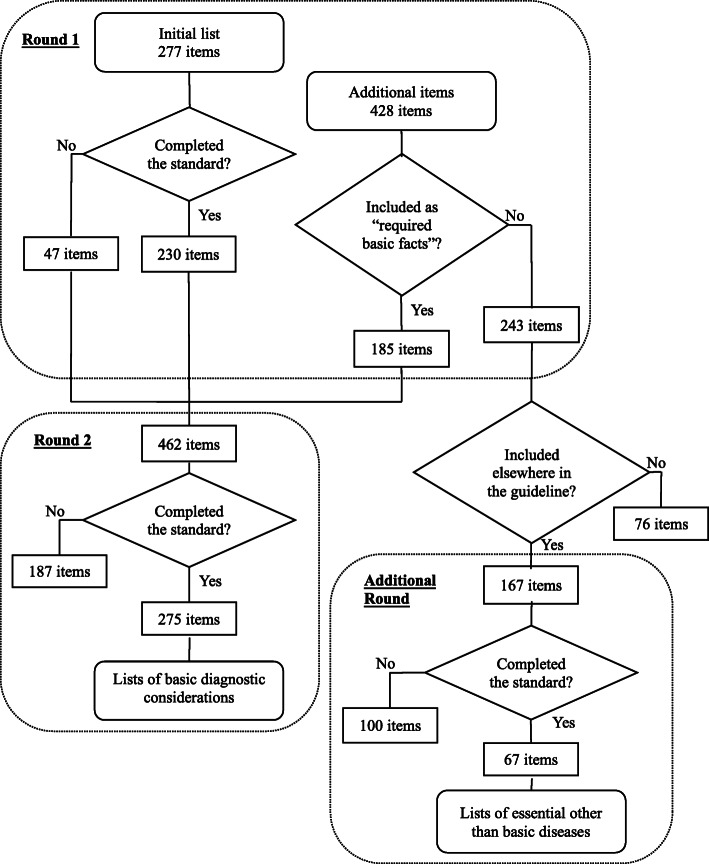
Numbers of items in the three rounds

In the first round, 47 items were eliminated from the initial lists (of 277 items) according to the pre-set standard. Among the 428 items that the study participants additionally suggested, 185 items that were also part of the “required basic facts” for the national licensing examination were included in the revised list for Round 2 (Fig. 1).

After analyzing the feedback our panel members gave in Round 2, we examined the face validity of all the lists on which consensus had been reached. This third version consisted of 13 items on average per signs, symptoms, or pathophysiology (and a median of 7 items) as basic diagnostic considerations for final-year medical students. In total, the final lists comprised 275 items; 187 items, including 79 items from the initial lists, were eliminated in the two rounds of the study.

In their free comments, some of the experts questioned the validity of the “required basic facts” for the national licensing exam being used as part of the standard. We decided to conduct one additional round for our panelists to evaluate the diseases they suggested in Round 1 that were not part of the “required basic facts” for the national licensing exam but were included elsewhere in the guideline. Among the 243 items that were added by the experts in Round 1, 76 were eliminated because they were not identified in the guideline. Among 167 items included in the guideline, 67 items that met the consensus standard were incorporated in lists of essential other than basic diseases. We obtained no complaints about the burden of answering the questionnaires from the panelists.

**Fig. 2:**
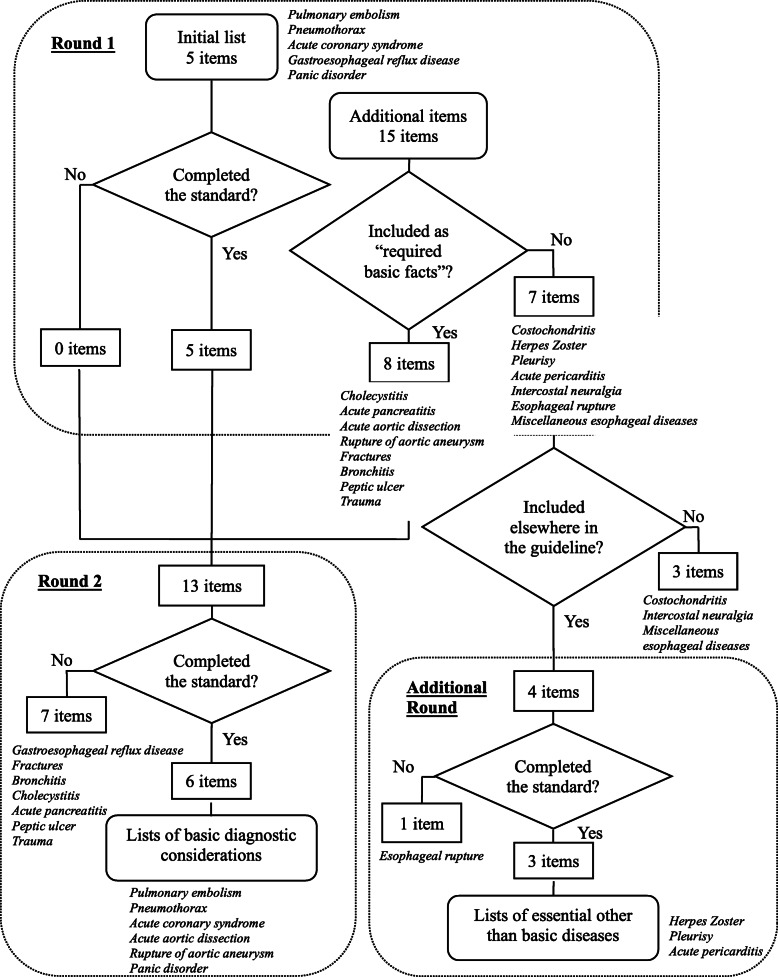
Evaluated items for “chest pain”

The final lists consisted of basic diagnostic considerations, and the lists of essential other than basic diseases are available as Additional file 1. As an example, the list of basic diagnostic considerations for the symptom of chest pain, as well as the lists of essential other than basic diseases are illustrated in Table 3. All items assessed in the three rounds for chest pain are shown in Fig. 2. Of the 23 participants who took part in the study, 22 completed the first and second rounds (96%), and 20 completed the additional round (87%).

**Table 3:** The example of “Chest pain”

Basic diagnostic considerations	Mean score (SD)	Number of experts who chose “must include” (%)
Respiratory		
Pulmonary embolism	4.6 (0.57)	21 (95)
Pneumothorax	4.7 (0.47)	22 (100)
Cardiovascular		
Acute coronary syndrome	4.9 (0.29)	22 (100)
Acute aortic dissection	4.9 (0.29)	22 (100)
Rupture of aortic aneurysm	4.8 (0.39)	22 (100)
Psychogenic		
Panic disorder	4.3 (0.75)	20 (91)
**Essential other than basic diseases**	**Mean score (SD)**	**Number of experts who chose “must include” (%)**
Acute pericarditis	4.1 (0.87)	15 (75)
Pleurisy	4.4 (0.57)	19 (95)
Herpes Zoster	4.0 (0.95)	15 (75)

Abbreviations: SD, Standard Deviation

## Discussion

This modified Delphi study identified 275 basic and 67 essential other than basic diseases as the potential diagnoses for 37 common signs, symptoms, and pathophysiology that Japanese medical students should master before graduation. The novelty of this study is that no similar study has been published so far, and we expect that the lists developed in the study could be transferable to other countries with a similar growing emphasis on patient safety and de-emphasis on hospital stay [32]. They can be useful for teaching and learning how to generate initial hypotheses because, as Bowen and ten Cate [9] suggest, a preselection of differential diagnoses to consider is crucial for novice learners acquiring this competence. Moreover, the method we adopted can be useful in other countries when developing a national consensus on lists of symptom-specific differential diagnoses across all medical disciplines.

The knowledge and skills regarding essential diseases for final-year medical undergraduates should be equivalent to those of new graduates. In the move toward competency-based medical education, Japan has been reforming competency-based postgraduate programs per the competencies developed during undergraduate medical education [33]. Thus, this research is significant because clinical faculty members and clinical teachers with experience in teaching both medical students and residents participated in the study. The results of our study can contribute to further revision of the national guideline for undergraduate medical education as well as stronger continuity between undergraduate and postgraduate education.

From the initial lists, 79 items were eliminated in the first two rounds of the study. Both Japanese and international literature were reviewed to develop the initial lists, and the clinical importance and urgency of diseases considered may be universal to some extent across time, countries, and ethnicity. However, these references do not necessarily include information reflecting the prevalence, social needs, and the possibility that Japanese learners experienced the disease in clinical rotations as medical students and in the workplace as residents immediately after graduation. For instance, “gastroesophageal reflux disease,” which was a component of the initial list for “chest pain” in the model core curriculum, was removed in the second round probably reflecting its prevalence of less than 5% in Asia compared to that of 10 to 20% in the Western world [34]. In addition, these were not necessarily materials that considered the level of medical students at graduation. The lack of such materials for undergraduate medical education is one of the rationales for this study. The study had experts with current information on contextual features not yet available in the literature who could create lists designed for medical students.

Moreover, the excluded 79 items included those that met the positive consensus standard in Round 1 but did not meet it in Round 2, after reevaluation in comparison with all the other candidate items, including the additional diseases referenced by the panelists. For example, as indicated in Fig. 2, “gastroesophageal reflux disease” met the standard in the first round, but was excluded in the second round when it was evaluated again with other items. This approach may cause lower response rates which could affect the validity of the study by making the lists longer [35]. However, the result suggests that this method can contribute to a more valid evaluation of items in Delphi studies. We believe that this method is appropriate for this study to inform diagnostic considerations for medical students since we had a relatively high response rate throughout the rounds, and the face validity of the lists specific to given signs, symptoms, and pathophysiology seems robust. Future studies can consider this method as well.

We anticipate that the derived lists will promote clinical reasoning education. For instance, even in traditional organ system-based preclinical curricula, they can be beneficial for developing case scenarios that consider a plausible set of differential diagnoses that overarch different organ systems. Students can learn how to generate initial hypotheses by comparing and contrasting the clinical features of those diseases, which may help them deepen their understanding of the pathophysiology by linking preclinical knowledge for each disease with experience in clinical rotations to develop an illness script for each disease. The lists will also support students and teachers in identifying pertinent information to differentiate those diseases when conducting medical interviews and physical examinations. As Yudkowsky et al. [36] state, checklists that exclusively assess discriminating information which affects diagnostic decisions have higher validity than those that assess thorough clinical features of each disease. Furthermore, as the use of artificial intelligence (AI) in the teaching of clinical reasoning has been increasing [37], the results of this study may be useful as a guideline regarding which diagnoses should be considered in machine learning. Similarly, the findings can also be beneficial in the development of clinical decision support systems.

However, we do not intend to encourage students to learn the diseases on the lists by heart. First, the lists are limited to the minimum required diseases of the national examination standards and do not cover all the diseases corresponding to the signs, symptoms, and pathophysiology. Suggesting memorization of the lists may cause the misconception that the listed diseases are sufficient, and adversely result in medical students’ mindless reasoning. Second, the resulting lists are the lists of potential diagnoses or diagnostic considerations related to each sign and symptom that medical students should master by the end of their undergraduate training, but not lists of differential diagnoses that should be examined at once in making diagnoses.

One of the study’s strengths is the high response rate across all three rounds of the Delphi process. According to Cantrill, Sibbald, and Buetow [38], response rates can influence the validity of the derived consensus. The high response rate may also indicate the perceived importance of this study to our panelists. Considering the limited reduction in respondent numbers after every round, we would argue that the results are valid. The validity of our study was also enhanced by ensuring a representative panel of experts recruited from a variety of institutions from across Japan.

This study also had some limitations. First, since this study was conducted in Japan alone, its international applicability may be limited. The study focused on Japanese curriculum requirements, and various epidemiological factors and societal needs should be considered in the interpretation and application of our results. Moreover, the resulting lists should change over time in accordance with changes of contextual features. Thus, the results of our study can be used as a baseline standard and should be tailored to match the specific educational needs of other countries.

Second, we limited the potential diagnoses to those listed in the “required basic facts” of the guideline for the national licensing examination and adopted a categorical representation of diseases according to the guideline. For example, when the panelists added “renal failure,” this was converted into “acute kidney disease” and “chronic kidney failure” as indicated in the guideline, and both were evaluated independently in the following rounds. Although the authors were aware of the possibility that this might eliminate some diseases that should be actively considered by final-year medical students, we opted to make the lists sufficiently compact to produce a consensus on the minimum requirements for final-year medical students, with a low variance in terminology. To reduce the possible influence of this limitation, we designed an additional round to evaluate items outside of the “required basic facts”.

Third, some of the signs, symptoms, and pathophysiology and their classifications might have confused our panelists. For instance, among the 37 common signs, symptoms, and pathophysiology considered in the model core curriculum, two different clinical conditions were combined into one condition several times, such as “Hematuria” and “Proteinuria,” “Anxiety” and “Depression,” and “Disturbance of consciousness” and “Syncope.” Some of the experts claimed that these were clinically distinct due to different but partly overlapping working diagnoses. Moreover, the panelists pointed out that the relationship between some independent signs, symptoms, and pathophysiology among the 37 was ambiguous, such as “Vertigo” and “Syncope.” They also reported a few irregular cases in which the concept of “clinical reasoning” did not seem to apply, such as cases of “Trauma” and “Burns.” These notes are crucial for future improvement of both the core medical curriculum and the guidelines for the national licensing examination.

Fourth, there are potential biases in the selection of the experts in our study. As for gender bias, no women agreed to participate. Women hold fewer academic positions [39] and have less research involvement, such as authorship on clinical reasoning [40], than men. In Japan, the proportion of female faculty in universities and clinical teachers in training hospitals is approximately 10% [41, 42]. The underrepresentation of women among the candidate panelists for this study reflects the gender difference in the academic positions in the Japanese medical society, although the first and corresponding author of this research (YM) is a female physician. Inviting more female experts in clinical reasoning education is essential for amplifying the voices of women.

In addition, clinical teachers from community hospitals were underrepresented, compared with those who belonged to universities and university hospitals. In medical education in Japan, clinical practice for undergraduate education is conducted mainly at university hospitals. Thus, as one condition for recruiting experts was that they were familiar with the model core curriculum for undergraduate medical education, we also made it a condition that teachers of community hospitals had an educational background as university teachers. As a result, the number of physicians at community hospitals may have decreased. We believe that this does not diminish the validity of the results in the Japanese setting since secondary hospitals are the main settings of undergraduate and postgraduate training in Japan. However, it is important to bear in mind this possible bias when interpreting the results in other countries.

Finally, initial differential diagnoses for signs, symptoms, or pathophysiology should be generated with a patient’s background information such as sex, age, and ethnicity, which were not considered in the lists generated in this study. We would advise future users of the lists to add such background information pertinent to differential diagnoses selected from the lists of diagnostic considerations.

## Conclusions

In conclusion, our study can contribute to promoting the integration of fragmented clinical reasoning teaching processes by encouraging students’ contrastive learning as well as ensuring greater continuity between undergraduate and postgraduate medical education in the Japanese setting. This is also important for other countries where the ever-expanding competencies required of medical students exacerbate the fragmentation of medical education [5]. The derived lists and the method employed for this study could apply to other countries. For our next step, we aim to develop a list of which medical history and physical examination elements final-year medical students are required to gather and master when considering the differential diagnoses on the lists resulting from this study.

## Supplementary Information

**Additional file 1.** The final lists consisted of basic diagnostic considerations and the lists of essential other than basic diseases as the potential diagnoses for 37 common signs, symptoms, and pathophysiology that Japanese medical students should master before graduation.

## Data Availability

The datasets used and analyzed during the current study are available from the corresponding author upon reasonable request.
