# Supplementary material for: Lists of potential diagnoses that final-year medical students need to consider: a modified Delphi study
Source: BMC Med Educ. 2021 Apr 23;21:234. doi: 10.1186/s12909-021-02652-5 (PMC8066856; doi:10.1186/s12909-021-02652-5)
Supplement: Supplementary file 1 — Additional file 1. The final lists consisted of basic diagnostic considerations and the lists of essential other than basic diseases as the potential diagnoses for 37 common signs, symptoms, and pathophysiology that Japanese medical students should master before graduation. [file 12909_2021_2652_MOESM1_ESM.docx]

**Lists of potential diagnoses that final-year medical students need to consider: A modified Delphi study**

Yuka Urushibara-Miyachi^1^, Makoto Kikukawa^2^, Masatomi Ikusaka^3^, Junji Otaki^4^, and Hiroshi Nishigori^1,5^

^1^Kyoto University, Kyoto, Japan, ^2^Kyushu University, Fukuoka, Japan, ^3^Chiba University Hospital, Chiba, Japan, ^4^Tokyo Medical University, Tokyo, Japan, ^5^Nagoya University, Nagoya, Japan

Correspondence should be addressed to Yuka Urushibara-Miyachi, Faculty of Medicine, Kyoto University, Yoshida konoe-cho, Sakyo-ku, Kyoto, 606-8501, Kyoto, Japan; telephone: +81-75-753-4300; e-mail: yuka.urushibara@gmail.com

**Additional file 1**

The final lists consisted of basic diagnostic considerations and the lists of essential other than basic diseases as the potential diagnoses for 37 common signs, symptoms, and pathophysiology that Japanese medical students should master before graduation.

1. Fever

| Basic diagnostic considerations | Essential other than basic diseases |
| --- | --- |
| **Infectious & Inflammatory**  Meningitis  Upper respiratory infection  　Tonsillitis  　Pneumonia  Tuberculosis  Acute sinusitis  Urinary tract infection  Cholecystitis  Cholangitis  **Intoxication**  Drug-induced | Influenza  Cellulitis  Infectious endocarditis |

1. General fatigue

| Basic diagnostic considerations | Essential other than basic diseases |
| --- | --- |
| **Infectious & Inflammatory**  Tuberculosis  Hepatitis  **Cardiovascular**  Heart failure  **Psychiatric**  Depression  **Endocrine & Metabolism**  Hypothyroidism  Iron deficiency anemia | None |

1. Appetite loss

| Basic diagnostic considerations | Essential other than basic diseases |
| --- | --- |
| **Digestive**  Peptic ulcer  Acute hepatitis  **Psychiatric**  Depression | Acute adrenal insufficiency |

4-1. Weight gain

| Basic diagnostic considerations | Essential other than basic diseases |
| --- | --- |
| **Acute**  Heart failure  Nephrotic syndrome  **Chronic**  Hypothyroidism | None |

4-2. Weight loss

| Basic diagnostic considerations | Essential other than basic diseases |
| --- | --- |
| **Neoplasms**  Malignancy in general  **Endocrine**  Diabetes  Hyperthyroidism  **Psychiatric**  Depression  **Respiratory**  Chronic obstructive pulmonary disease | Anorexia nervosa |

1. Shock

| Basic diagnostic considerations | Essential other than basic diseases |
| --- | --- |
| **Hypovolemic**  Acute gastrointestinal bleeding  Rupture of aortic aneurism  **Cardiogenic**  Acute coronary infarction  **Obstructive**  Tension pneumothorax  Pulmonary embolism  **Distributive**  Sepsis  Anaphylaxis | Cardiac tamponade  Acute adrenal insufficiency |

1. Heart arrest

| Basic diagnostic considerations | Essential other than basic diseases |
| --- | --- |
| **Cardiovascular**  Acute coronary infarction  Arrhythmia  Acute aortic dissection  Rupture of aortic aneurysm  **Respiratory**  Pulmonary embolism  Tension pneumothorax  Traumatic pneumothorax  **Neurogenic**  Subarachnoid hemorrhage  Cerebral hemorrhage  **Autoimmune**  Anaphylaxis  **Intoxication**  Drug intoxication | Cardiac tamponade  Abnormalities of potassium metabolism |

1. Disturbance of consciousness/syncope

| Basic diagnostic considerations | Essential other than basic diseases |
| --- | --- |
| **Central nervous system**  Subarachnoid hemorrhage  Intracranial hematoma  Encephalitis  Cerebral hemorrhage  Cerebral infarction  Meningitis  Drug intoxication  Alcoholic intoxication  **Systemic**  Epilepsy  Cardiac infarction  Arrhythmia  Valvular disease (Aortic valvular disease)  Acute aortic dissection  Pulmonary embolism  Acute gastrointestinal bleeding  Sepsis | Hypoglycemia  Shock  Carbon dioxide narcosis  Abnormalities of sodium metabolism |

1. Seizure

| Basic diagnostic considerations | Essential other than basic diseases |
| --- | --- |
| **Cerebrovascular**  Cerebral infarction  Cerebral hemorrhage  **Infectious**  Encephalitis  Encephalopathy  Febrile convulsion  **Idiopathic**  Epilepsy | None |

1. Dizziness

| Basic diagnostic considerations | Essential other than basic diseases |
| --- | --- |
| **Peripheral**  Benign paroxysmal positional vertigo  **Central**  Cerebral hemorrhage  Cerebral infarction | Meniere’s disease  Vestibular neuronitis |

1. Dehydration

| Basic diagnostic considerations | Essential other than basic diseases |
| --- | --- |
| **Digestive**  Acute enteritis  Infantile diarrhea  **Environmental**  Heatstroke | None |

1. Edema

| Basic diagnostic considerations | Essential other than basic diseases |
| --- | --- |
| **Local**  Deep venous thrombosis  **Systemic**  Heart failure  Nephrotic syndrome  Chronic kidney disease  Liver cirrhosis  Hypothyroidism  Drug-induced | Lymphedema  Angioedema |

1. Rash

| Basic diagnostic considerations | Essential other than basic diseases |
| --- | --- |
| **Infectious**  Measles  Rubella  Varicella  Herpes  **Allergic & Autoimmune**  Hives  Drug eruption  Atopic dermatitis  **Other**  Eczema | Erythema nodosum  Erythema infectiosum  Herpes zoster |

1. Cough/sputum

| Basic diagnostic considerations | Essential other than basic diseases |
| --- | --- |
| **Infectious**  Upper respiratory infection  Sinusitis  Bronchitis  Pneumonia  Pulmonary tuberculosis  **Neoplasms**  Lung cancer  **Idiopathic**  Interstitial lung disease  **Intoxication**  Drug-induced  **Autoimmune**  Bronchial asthma  Allergic rhinitis  **Digestive**  Gastroesophageal reflux disease | Common cold syndrome  Pertussis |

1. Hemosputum/hemoptysis

| Basic diagnostic considerations | Essential other than basic diseases |
| --- | --- |
| Pulmonary tuberculosis  Lung cancer | Bronchiectasis |

1. Dyspnea

| Basic diagnostic considerations | Essential other than basic diseases |
| --- | --- |
| **Respiratory**  Pulmonary embolism  Acute respiratory distress syndrome  Bronchial asthma  Chronic obstructive pulmonary disease  Pneumonia  Interstitial pulmonary disease  Pulmonary tuberculosis  Tension pneumothorax  Spontaneous pneumothorax  **Cardiovascular**  Heart failure  **Allergic**  Anaphylaxis | Acute epiglottitis  Suffocation |

1. Chest pain

| Basic diagnostic considerations | Essential other than basic diseases |
| --- | --- |
| **Respiratory**  Pulmonary embolism  Pneumothorax  **Cardiovascular**  Acute coronary syndrome  Acute aortic dissection  Rupture of aortic aneurysm  **Psychogenic**  Panic disorder | Acute pericarditis  Pleurisy  Herpes Zoster |

1. Palpitation

| Basic diagnostic considerations | Essential other than basic diseases |
| --- | --- |
| **Cardiovascular**  Arrhythmia  **Secondary**  Hyperthyroidism  Iron deficiency anemia  Secondary anemia  **Psychogenic**  Panic disorder  Anxiety disorder | None |

1. Pleural effusion

| Basic diagnostic considerations | Essential other than basic diseases |
| --- | --- |
| **Cardiovascular**  Heart failure  **Respiratory**  Pneumonia  Pulmonary tuberculosis  Lung cancer  **Digestive**  Liver cirrhosis | Pleurisy  Pleural empyema |

1. Dysphagia

| Basic diagnostic considerations | Essential other than basic diseases |
| --- | --- |
| **Nervous**  Cerebral hemorrhage  Cerebral infarction  **Respiratory**  Tonsillitis  **Digestive**  Esophageal cancer | None |

1. Abdominal pain

| Basic diagnostic considerations | Essential other than basic diseases |
| --- | --- |
| **Digestive**  Peptic ulcer  Functional dyspepsia  Acute gastroenteritis  Acute appendicitis  Constipation  Diffuse peritonitis  Irritable bowel syndrome  Intestinal obstruction  Intussusception  Inguinal hernia  Cholecystitis  Cholelithiasis  Acute pancreatitis  **Cardiovascular**  Acute coronary syndrome  Rupture of (abdominal) aortic aneurysm  Acute aortic dissection  **Urogenital**  Abortion & premature labor  Ovarian cyst (with torsion)  Ovarian cancer (with torsion)  Endometriosis  Ureteral stone | Diverticulitis  Ischemic colitis  Mesenteric artery thrombosis  Ectopic pregnancy  Diabetic ketoacidosis |

1. Nausea/vomit

| Basic diagnostic considerations | Essential other than basic diseases |
| --- | --- |
| **Digestive**  Acute gastroenteritis  Acute appendicitis  Intestinal obstruction  Food intoxication  **Nervous**  Cerebral hemorrhage  Migraine  Subarachnoid hemorrhage  Intracranial hematoma  Meningitis  **Cardiovascular**  Acute coronary syndrome  **Endocrine**  Pregnancy | Diabetic ketoacidosis  Abnormalities of calcium metabolism |

22-1. Hematemesis

| Basic diagnostic considerations | Essential other than basic diseases |
| --- | --- |
| **Esophagus**  Esophageal varix  **Stomach**  Peptic ulcer  Gastric cancer | Mallory-Weiss syndrome |

22-2. Melena

| Basic diagnostic considerations | Essential other than basic diseases |
| --- | --- |
| **Upper gastrointestinal**  Peptic ulcer  **Lower gastrointestinal**  Inflammatory bowel disease  Colon cancer  Hemorrhoid | Anal fissure  Ischemic colitis  Diverticular hemorrhage |

23-1. Constipation

| Basic diagnostic considerations | Essential other than basic diseases |
| --- | --- |
| **Functional**  Constipation  Irritable bowel syndrome  Hypothyroidism  Drug-induced  Parkinson disease  **Organic**  Intestinal obstruction  Colon cancer | None |

23-2. Diarrhea

| Basic diagnostic considerations | Essential other than basic diseases |
| --- | --- |
| **Inflammatory**  Acute gastroenteritis  Inflammatory bowel disease  **Abnormalities of bowel movement**  Irritable bowel syndrome  Hyperthyroidism  **Iatrogenic**  Drug-induced | None |

24. Jaundice

| Basic diagnostic considerations | Essential other than basic diseases |
| --- | --- |
| **Conjugated**  Acute hepatitis  Chronic hepatitis  Liver cirrhosis  Hepatic cancer  Cholangitis  Pancreatic cancer  Drug-induced  **Unconjugated**  Hemolytic anemia  Drug-induced | Cholangiocarcinoma  Physiological neonatal jaundice |

25. Abdominal distension/mass

| Basic diagnostic considerations | Essential other than basic diseases |
| --- | --- |
| **Digestive**  Intestinal obstruction  Inguinal hernia  **Ascites**  Liver cirrhosis  **Other**  Pregnancy | None |

26. Anemia

| Basic diagnostic considerations | Essential other than basic diseases |
| --- | --- |
| **Iron deficiency anemia**  Peptic ulcer  Uterine myoma  **Hematopoietic neoplasms**  Leukemia  Myeloma  **Secondary anemia**  Chronic kidney disease | Vitamin B12 deficiency, Pernicious anemia  Folic acid deficiency anemia  Hemolytic anemia  Myelodysplastic syndromes  Menorrhagia |

27. Lymphadenopathy

| Basic diagnostic considerations | Essential other than basic diseases |
| --- | --- |
| **Infectious**  Tonsillitis  Rubella  Tuberculosis  **Neoplasms**  Malignant lymphoma  Other malignancies in general | Infectious mononucleosis |

28-1. Abnormality of urine and urination: polyuria

| Basic diagnostic considerations | Essential other than basic diseases |
| --- | --- |
| **Osmotic diuresis**  Diabetes  **Intoxication**  Drug-induced | None |

28-2. Abnormality of urine and urination: frequent urination

| Basic diagnostic considerations | Essential other than basic diseases |
| --- | --- |
| **Storage dysfunction**  Urinary tract infection  **Voiding dysfunction**  Prostatic hyperplasia | Overactive bladder  Neurogenic bladder |

29. Hematuria/proteinuria

| Basic diagnostic considerations | Essential other than basic diseases |
| --- | --- |
| **Kidney**  Glomerulonephritis syndrome  Nephrotic syndrome  Diabetic kidney disease  Renal cell carcinoma  **Urinary tract & Bladder**  Ureteral stone  Urinary tract infection  Bladder cancer | None |

30. Menstrual disorders

| Basic diagnostic considerations | Essential other than basic diseases |
| --- | --- |
| **Amenorrhea**  Pregnancy  Drug-induced  **Dysmenorrhea**  Primary dysmenorrhea  Endometriosis  **Metrorrhagia**  Uterine cancer  Menopausal disorders | None |

31. Anxiety/depression

| Basic diagnostic considerations | Essential other than basic diseases |
| --- | --- |
| **Primary**  Depression  Bipolar disorder  Anxiety disorder  **Secondary anxiety disorder**  Hyperthyroidism  Malignancy in general  **Secondary depression**  Dementia  Parkinson’s disease  Hypothyroidism  Malignancy in general  Drug-induced | Adjustment disorder |

32. Memory loss

| Basic diagnostic considerations | Essential other than basic diseases |
| --- | --- |
| **Vascular**  Cerebral infarction  **Degenerative**  Dementia  Parkinson’s disease  **Psychiatric**  Depression  **Endocrine & Metabolism**  Hypothyroidism  **Iatrogenic**  Drug-induced | Normal pressure hydrocephalus  Chronic subdural hematoma |

33. Headache

| Basic diagnostic considerations | Essential other than basic diseases |
| --- | --- |
| **Functional**  Tension headache  Migraine  Drug-induced  **Symptomatic**  Meningitis  Cerebral hemorrhage  Subarachnoid hemorrhage  Glaucoma  Acute sinusitis | Cluster headache  Giant cell arteritis (temporal arteritis) |

34. Motor paralysis

| Basic diagnostic considerations | Essential other than basic diseases |
| --- | --- |
| **Brain**  Cerebral infarction  Transient ischemic attack  Cerebral hemorrhage  Intracranial hematoma  Epilepsy  **Spinal cord**  Spinal cord injury  Disc herniation | Polymyositis  Dermatomyositis  Amyotrophic lateral sclerosis  Guillain-Barré syndrome  Abnormalities of potassium metabolism |

35. Back pain

| Basic diagnostic considerations | Essential other than basic diseases |
| --- | --- |
| **Cardiovascular**  Acute aortic dissection  **Digestive**  Acute pancreatitis  Pancreatic cancer  **Urogenital**  Ureteral stone  **Musculoskeletal & Spinal cord**  Disc herniation  Spondylosis deformans  Spinal canal stenosis  Vertebral compression fracture | Acute low back pain  Pyogenic spondylitis |

36. Arthralgia/swollen joint

| Basic diagnostic considerations | Essential other than basic diseases |
| --- | --- |
| **Monoarthritis**  Gout  Trauma  **Polyarthritis**  Rheumatoid arthritis  Systemic lupus erythematosus | Pseudogout  Reactive arthritis  Septic arthritis |

37. Trauma/burn

| Basic diagnostic considerations | Essential other than basic diseases |
| --- | --- |
| **Primary**  Head injuries  Fracture  Traumatic pneumothorax  Burn  **Secondary**  Cerebral hemorrhage  Subarachnoid hemorrhage  Intracranial hematoma  Acute aortic dissection | None |
